# The ameliorative potentials of aqueous leaf extract of *Amaranthus hybridus* on monosodium glutamate-induced testicular damage in Wistar rats

**DOI:** 10.5935/1518-0557.20210091

**Published:** 2022

**Authors:** David O Oyeniran, Abdulfatai O Ojewale, Adebanji M Akingbade, Babatunde J Dare, Mario A Adelaja, Latifat O Oseni

**Affiliations:** 1 Department of Anatomy, Faculty of Basic Medical Sciences, Lagos State University College of Medicine, Ikeja, Lagos, Nigeria; 2 Department of Anatomy, Faculty of Basic Medical Sciences, Ekiti State University College of Medicine, Ado-Ekiti, Ekiti, Nigeria; 3 Department of Anatomy, Faculty of Basic Medical Sciences, Osun State University College of Health Sciences, Osogbo, Osun, Nigeria; 4 Anatomic Pathology Unit, LagPath Laboratory, Ikeja, Lagos, Nigeria; 5 Training Schools Education Research and Statistics (TSERS) Department, National Orthopaedic Hospital, Igbobi, Lagos, Nigeria

**Keywords:** monosodium glutamate, Amaranthus hybridus, rat, testicular architecture and infertility

## Abstract

**Objective:**

*Amaranthus hybridus* (AH) is a food plant commonly eaten in our country known as a good source of vitamins, minerals, and antioxidants. The present study was designed to investigate the ameliorative potentials of aqueous extract of A. hybridus on Monosodium Glutamate (MSG) -induced testicular toxicity in adult Wistar rats.

**Methods:**

Thirty-two male Wistar rats weighing 160-180 g were divided into four groups. Group A served as control; rats in Group B were given 300 mg/kg of body weight (BW) of aqueous leaf extract of AH; rats in Group C were given 4 mg/g (BW) of 40% MSG; and rats in Group D were given 4 mg/g (BW) of 40% MSG and 300 mg/kg (BW) of extract orally for 6 weeks.

**Results:**

There was a significant increase in body weight and a significant reduction in testis weight, testis volume, and testis/body weight ratio in the group given only MSG when compared with controls. Histologically, rats in Groups A and B had normal testicular architecture, while the rats given MSG only showed a significant derangement in testicular histoarchitecture and impaired sperm parameters when compared with controls and the rats given AH. However, these derangements were alleviated in the MSG+AH group when compared with controls.

**Conclusions:**

Aqueous leaf extract of AH ameliorated the testicular derangement resulting from MSG administration.

## INTRODUCTION

Researchers have reported on the composition and functional properties of domestic and edible wild plants used in traditional medicine in developing countries (Oliveira & de Carvalho, 1975; Özbucak et al., 2007; Nsimba *et al*., 2008). *Amaranthus hybridus* is a food plant that belongs to the Amaranthaceae family popularly called "Amaranth or Pigweed" in English, "Efotete" by the Yorubas, "Inine" by the Igbos, and "Alayyafoo" by the Hausas. It is an annual herbaceous plant that grows to 1-6 feet high. The leaves are alternate petiole, 3-6 inches long, dull green, and rough, hairy, ovate or rhombic with wavy margins. In most developing countries the leaves of A. hybridus are used to prepare soups, and eaten as spinach or salad (Martin & Telek, 1978; Mepba *et al*., 2007). It has been reported that A. *hybridus* contains protein, carotenoids, thiamine, riboflavin, niacin, pyridoxine, α-tocopherol, flavonoids, polyphenol, vitamins C and E, and minerals such as calcium, iron, zinc, magnesium, and phosphorus, which makes it a great source of antioxidants (Özbucak et al., 2007; Shukla *et al*., 2006; Akubugwo *et al*., 2007; Ouedraogo *et al*., 2011). In traditional medicine, the plant is used as a digestive, laxative, diuretic, antipyretic agent. It improves appetite, liver infections, and knee pain (Nacoulma, 1996). It has been reported that plant extracts are used as vermifuge (Nacoulma, 1996). The aqueous extracts of *A. hybridus* showed anti-anemic activity activity on rabbits treated with phenyl hydrazine hydrochloride (Ogbe *et al*., 2010).

Monosodium glutamate (MSG) is a well-known food additive. It is highly hydrophilic due to the hydroxyl group in its structure. It is easy to find and cheap (Samuels, 1999). MSG is one of the acidic class amino acids that, though not considered essential, is operant in healthy human metabolism (Samuels, 1999). It is widely distributed in protein foods and even some plant proteins yield as much as 45% of their weight as glutamic acid. Glutamic acid is a naturally occurring amino acid in many proteins. Rich sources of glutamic acid are soya, meat, poultry, fish, eggs, and dairy products (IFIC, 1994). Biodun & Biodun (1993) reported that MSG was toxic to human and experimental animals and might produce symptoms such as burning sensations in the back of the neck, forearms, chest, facial pressure/tightness, chest pain, headache, nausea, palpitation, numbness in the back of neck radiating to arms and back, tingling, warmth, weakness in face, temples, upper back, neck, and arms, bronchospasm (observed in asthmatics only), drowsiness, and weakness. MSG has a toxic effect on the testis by causing significant oligozoospermia and increases abnormal sperm morphology in a dose-dependent fashion in male Wistar rats (Giovambattista *et al*., 2003; Nayanatara *et al*., 2008).

Several studies have reported the implication of MSG in cases of male infertility as it causes testicular disruption, deterioration, and change of sperm cell population and morphology (Oforofuo *et al*., 1997; Eweka, 2006). There has also been a report of MSG damaging nerve cells of the hypothalamus, affecting the hypothalamic-pituitary-gonadal regulatory axis (Igwebuike *et al*., 2011), and inducing oxidative stress (Singh *et al*., 2003; Diniz *et al*., 2004). MSG-induced organ cytotoxicity has been shown to be through the direct effect, oxidative pathway and neurotoxic effect. This study was designed to find whether A. hybridus might ameliorate testicular toxicity and the spermatogenic effects caused by MSG.

## MATERIALS AND METHODS

### Materials

#### Chemicals

Monosodium glutamate (MSG) (Ajinomoto^®^) was purchased from the Amazing Grace Stores, Ikeja, Lagos, Nigeria.

#### Collection and authentication of the plant

*A. hybridus* leaves were purchased from a local market in Ikotun, Lagos, Nigeria and were identified and authenticated in the Forestry Research Institute of Nigeria (FRIN), Ibadan, Nigeria. A voucher specimen was deposited in the herbarium and assigned a voucher number (FHI 703215)

#### Experimental Animals

The 32 Male Wistar rats weighing between 160g to 180g used in this study were obtained from a breeding stock maintained in the Animal House of the Lagos State University College of Medicine (LASUCOM), Ikeja, Lagos State. They were housed under standard laboratory conditions with a 12-hour daylight cycle and had access to feed and water *ad libitum*. They were acclimatized to laboratory conditions for two weeks before the commencement of the experiments. Approval for the use of animals was sought and obtained from the Departmental Ethics Committee on Animal Use. The weights of the animals were estimated at procurement, during acclimatization, at commencement of the experiment, and twice within a week throughout the duration of the experiment, using an electronic analytical precision balance (BA210S, d=0.0001g) (Satorious GA, Goettingen Germany).

Experimental procedures involving the animals and their care was conducted in conformity with International, National and Institutional guidelines for the care of laboratory animals in Biomedical Research and use of laboratory Animals in Biomedical Research as promulgated by the Canadian Council of Animal Care (CCAC, 1993).

### Method

#### Plant aqueous extract

The fresh leaves of A. hybridus were thoroughly washed in clean sterile water. The water was then drained from the leaves; the leaves were then air-dried and grounded into a powder using mortar and pestle. A measure of 100g of the powder was dissolved in 1000 ml of boiled distilled water. It was then centrifuged at 4000 rpm at 4oC for 10 minutes. Then the supernatant part was evaporated to dryness in an oven at 40oC±5oC. The dry extract (12.70 g) was stored in a refrigerator at 4°C. A measure of 1 g of the dried extract was dissolved in 20 ml of distilled water to make an aqueous extract with a concentration of 50 mg/ml, which was used in this study.

#### Animal grouping and treatment

The rats were randomly divided into four groups (A, B, C and D) consisting of eight (8) animals each and were housed in four cages in such a way that the average weight difference between and within groups did not exceed ± 20% of the average weight of the sample population

Rats in Group A served as controls and were fed with normal diet. Group B rats were given 300 mg/kg of body weight of *A hybridus* aqueous leaf extract; Group C rats were given 4 mg/g of body weight of 40% monosodium glutamate; and Group D rats were given 4 mg/g of body weight of 40% monosodium glutamate and 300 mg/kg of body weight of the extract. Administration was performed orally using an oral gavage tube daily for 6 weeks.

#### Animal sacrifice and sample collection

At the time of sacrifice, the rats were weighed and then anesthetized with diethyl ether. The abdominal cavity was opened up through a midline abdominal incision to expose the reproductive organs. Then the testes and epididymides were excised. The testes of each animal were weighed with an electronic sensitive analytical balance (BA 210S, d=0.0001- Sartoriusen GA, Goettingen, Germany). The volume of each testis was measured by the water displacement method. The two testes of each rat were measured and the average value obtained for each of the two parameters was regarded as one observation. The testis of each animal was fixed in Bouin's solution for histological examination.

#### Sperm motility and progressive motility

As described by Panjeh Shahin *et al*. (2005), the caudal part of the epididymis was separated from the testis and placed in a beaker containing 1ml buffered physiological saline solution; then the section was incised with a pair of sharp scissors and left for a few minutes to liberate its spermatozoa into the saline solution. Semen drops were placed on the slide and two drops of warm 2.9% sodium citrate were added. The slide was covered with a cover slip and examined under the microscope under 400x magnification.

#### Total sperm count

Sperm counts were determined using a new improved Neubauer counting chamber (hemocytometer). One drop of the diluted sperm suspension was transferred to each counting chamber of the hemocytometer and allowed to stand for 5 minutes. The chamber was placed under a binocular light microscope using an adjustable light source. The ruled part of the chamber was then focused and the spermatozoa were counted in four 16-celled squares. Sperm concentration was calculated and expressed as [x] x 10⁶/ml, where x is the number of spermatozoa in a 16-celled square.

#### Sperm morphology

Sperm cell morphology was evaluated with the aid of a light microscope at 400x magnification. Caudal sperm was taken from the original dilution for motility and diluted 1:20 in 10% neutral buffered formalin (Sigma-Aldrich, Oakville, ON, Canada). Five hundred spermatozoa from the sample were scored for morphological abnormalities (Ateşşahin *et al*., 2006). Spermatozoa with a rudimentary tail, round head, or detached head were considered as morphologically abnormal.

#### Histological procedures

At the end of the experimental period, the animals were sacrificed. The testes were excised and fixed with Bouin's solution for 24 hours. Later, they were dehydrated in graded concentrations of ethanol, cleared in xylene, and embedded in paraffin wax in an oven at 57°C. The sections were cut in 5-µm thick slices, mounted on glass slides, and stained with hematoxylin and eosin and mounted with DPX. The sections of the testes were examined on a light microscope at 400x magnification.

#### Statistical analysis

All quantitative data were expressed as mean ± SD of number of experiments (n=8). The level of homogeneity among the groups was tested using Analysis of Variance (ANOVA) according to Snedecor & Cochran (1980). A value of *p*<0.05 was considered to indicate a significant difference between groups (Duncan, 1957). Analysis of data was done using an electronic calculator and the Statistical Package for Social Sciences (SPSS)/ PC computer program (version 16.0 SPSS).

## RESULTS

### Body weight, testis weight and testis volume

Table 1 shows that the rats in Groups A and B had a normal growth rate; rats in Group C had a significant increase in body weight when compared with controls (*p*<0.05); and rats in Group D were not significantly different from controls. At *p*<0.05 Rats in Group C (given only MSG) had a significant reduction in testis weight, volume, and testis/body weight ratio when compared with controls, as shown in Table 1.

**Table 1.** Effects of monosodium glutamate and *A.hybridus* on gross anatomical parameters.

Parameters	GROUP A	GROUP B	GROUP C	GROUP D
Initial Body Weight (g)	170±7.9	168.0±2.7	175.0±5.0	166.0±8.2
Final Body Weight (g)	199.0±11.4	198.0±5.7	234.0±96*	210.0±23.5
Body Weight (g)	29.0±14.3	30.0±7.0	59.0±12.4*	50.0±13.7
Testis Weight Diff (g)	1.44±0.1	1.42±0.1	1.0±0.1	1.3±0.1
Testis Volume (mL)	1.30±0.1	1.30±0.0	0.8±0.1*,**	1.2±0.1
Body Weight / Body Weight ratio	0.0007	0.0007	0.004*,**	0.006

* *p*<0.05 and

** *p*<0.001 significantly different from controls.

Values are expressed as mean ± SD for n=8 in each group.

### Epididymal sperm characteristics

#### Sperm count and sperm motility

The rats given only MSG had a significant reduction in sperm count and sperm motility (*p*<0.05) when compared controls and rats given AH. Rats given MSG and AH had no significant difference in sperm count and sperm motility when compared with controls (Table 2).

**Table 2.** Effects of monosodium glutamate and *Amaranthus hybridus* on sperm parameters.

Parameters	GROUP A	GROUP B	GROUP C	GROUP D
Sperm Count (x10^6^/ml)	159.4±46.2	168.0±47.1	79.0±19.2*	158.0±43.2
Sperm Motility	70.0±7.0	78.0±8.4	49.0±7.1*	68.0±4.5
Sperm Progressivity	a^1^	a^1^	b^1^*	b^1^
Sperm with normal morphology (%)	74.0±11.4	78.0±13.0	42.0±19.2*	68.0±8.4
Sperm with abnormal morphology (%)	26.0±11.4	22.0±13.0	58.0±19.2*	32.0±8.4

* *p*<0.05; significantly different from controls.

Values are expressed as mean ± SD for n=8 in each group.

a_1_ = rapid linear progressive motility,

b1 = slow sluggish linear or non-linear motility.

#### Sperm progressive motility and morphology

Rats in Groups A and B had no significant difference in sperm progressive motility and morphology (*p*<0.05). Rats given MSG only showed a significant (*p*<0.05) reduction in progressive motility and normal sperm morphology rates and a corresponding (*p*<0.05) significant increase in abnormal sperm morphology. Rats treated with AH had normal sperm progressive motility and morphology (Table 2).

#### Histology

**Figure 1:**
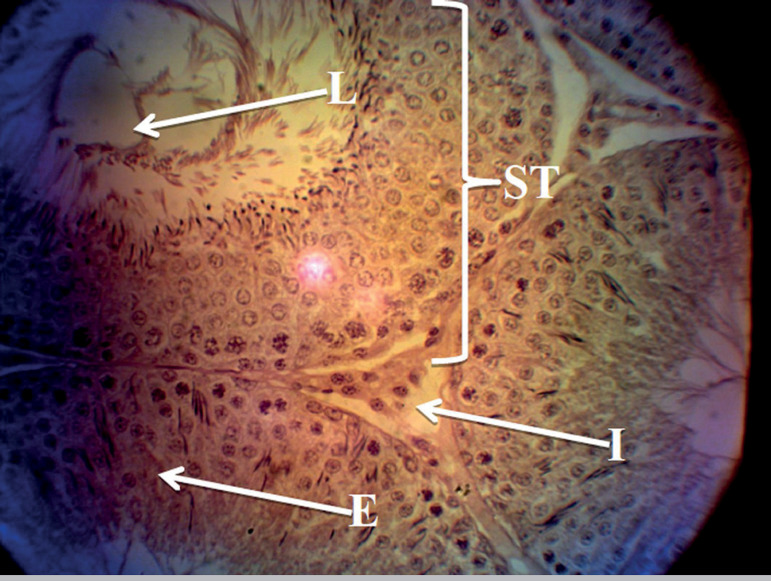
Histology of the testis of the control group showing normal testicular histology with intact seminiferous tubules (ST), interstitium containing leydig cell (I), seminiferous epithelium containing the spermatogenic cells and sertoli cells (E) and seminiferous lumen (L). H&E stain; (X400).

**Figure 2:**
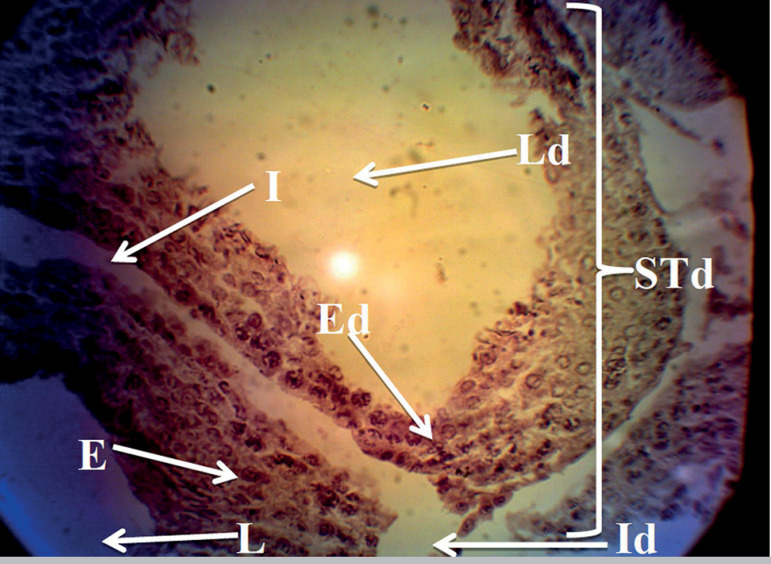
Histology of the testis of rat treated with AH only showing normal testicular histology with intact seminiferous tubules (ST), interstitium containing leydig cell (I), seminiferous epithelium containing the spermatogenic cells and sertoli cells (E) and seminiferous lumen (L). H&E stain; (X400).

**Figure 3:**
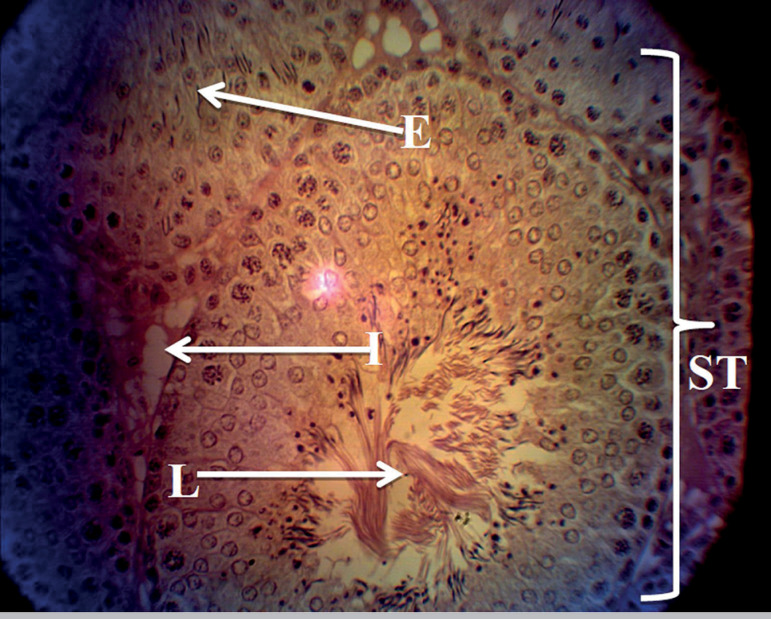
Histology of the testis of rat treated with MSG only showing a distorted testicular histology characterized by distorted seminiferous tubules (STd), damaged interstitium (Id), degenerated epithelium (Ed) and lumen devoid of spermatozoa (Ld). interstitium (I), seminiferous epithelium (E) and seminiferous lumen (L) H&E stain;(X400).

**Figure 4:**
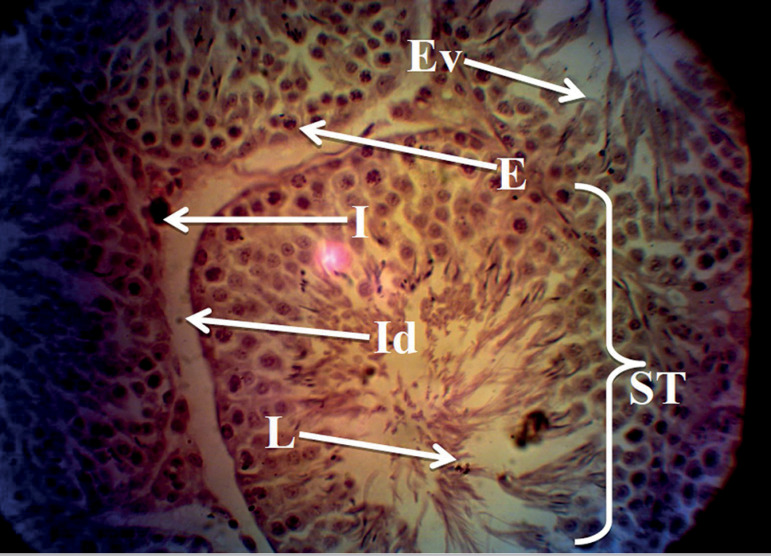
Histology of the testis of rat treated with MSG and AH showing fairly normal testicular histology with little degeneration of the interstitium (Id) and vacuolization of the seminiferous epithelium (Ev). Seminiferous tubules (ST), interstitium (I), seminiferous epithelium (E) and seminiferous lumen (L). H&E stain; (X400).

The histology profiles of rats in Groups A and B were normal with intact seminiferous tubules, interstitium, and numerous spermatozoa in the lumen. The seminiferous epithelium was densely packed with cells (Figure 1 and 2), while rats given only MSG showed a derangement in the histoarchitecture of the testis characterized by distorted seminiferous tubules, damaged interstitium, lumen devoid of spermatozoa, degeneration of seminiferous epithelium and tubules (Figure 3). All these derangements were ameliorated in the Group treated with MSG and AH with little vacuolization in the seminiferous epithelium (Figure 4).

## DISCUSSION

Male infertility accounts for up to half of all cases of infertility. In the general population, 1 in 20 men are infertile (McLachlan & de Kretser, 2001). In male infertility, semen is the target in diagnosis, therapeutic interventions, and analysis. Evidence suggests that male infertility is caused by either disturbances in testicular morphology, testicular function, endocrine function, biochemical activities or nuclei/chromosomal formation of the testicular cells. These lead to decreased sperm count and motility, testicular necrosis, testicular apoptosis, testicular atrophy or increases in the incidence of sperm with abnormal morphology, which may result in male infertility. Monosodium glutamate (MSG), one of the main flavor enhancers used in food products, is known to affect the structure and function of the male reproductive system and potentially cause infertility in male experimental animals (Das & Ghosh, 2010; Egbuonu *et al*., 2009).

In our study, the rats treated with MSG had significant increases in body weight when compared with controls (*p*<0.05). The increase in rat body weight seen in our study is in accordance with the findings published by Onyema *et al*. (2006); Inuwa *et al*. (2011); Nosseir *et al*., (2012) and Hummadi (2012), which reported that MSG influenced the appetite positively and induced weight gain. 

The rats treated with MSG + AH and AH only were not significantly different (*p*<0.05) from controls in terms of organ weight, testis volume, testis weight/body weight ratio; the Group treated with MSG only presented significant reductions in these parameters when compared with controls (*p*<0.05) (Table 1). Nosseir *et al*. (2012) also found that the testes of rats given MSG were smaller than the testes of controls. Nayanatara *et al*. (2008) and Oforofuo *et al*. (1997) also described MSG-induced decreases in testicular weight.

In terms of histology, our study found that rats given MSG only presented vacuolization of the interstitium and hypospermatozoa formation in the seminiferous tubules with lumen devoid of spermatozoa and significant reduction of the basal seminiferous epithelial cells (Figure 3). The histological evidences described in this study are consistent with several other reports on male Infertility regarding MSG administration (Oforofuo *et al*., 1997; Ismail, 2012; Alalwani, 2014). This may result from a direct effect of MSG or a neurotoxic effect on the hypothalamus-pituitary-gonadal axis affecting normal testicular function leading to infertility (Ismail, 2012; França *et al*., 2006). These derangements were not found in the Group given MSG and AH (Figure 4). This might result from the antioxidant properties of AH in savaging the free radicals potentially generated by MSG that may produce tissue damage.

Rats administered MSG have been reported to present sperm parameter decreases (Nayanatara *et al*., 2008; Oforofuo *et al*., 1997; Nosseir *et al*., 2012). A significant decline in sperm count was indicated in the Group given MSG only. Our results are in agreement with other reports (Giovambattista *et al*., 2003; Nayanatara *et al*., 2008; Igwebuike *et al*., 2011). Concomitant treatment with AH averted the derangement in sperm count and yielded values comparable to those seen in controls. This might result from the antioxidant constituents of *A.hybridus* (α-tocopherol, ascorbic acid, flavonoid), which probably scavenged the reactive oxygen species (free radicals) potentially generated by MSG that would otherwise produce sperm cell damage. Along the same lines, Ekaluo *et al*. (2013) found that vitamin C - an antioxidant - protected sperm cells against toxicity induced by MSG in rats. The Group given MSG only had significant increases (*p*<0.05) in sperm with abnormal morphology when compared with controls, as similarly described by Oforofuo *et al*. (1997), Nosseir *et al*. (2012) and Ismail (2012). The proportions of normal and abnormal sperm morphology in the rats treated with MSG and AH were not significantly different from the values seen in controls (Table 2). Sperm motility in Group C was significantly (*p*<0.05) reduced when compared with controls, while Group D was not significantly different from controls (Table 2). Progressive motility in rats treated with MSG and AH was significantly (*p*<0.05) higher than in the Group given MSG only (Table 2). The present result agrees with previous studies in which animals treated with MSG were found to have a reduction in caudal epididymal sperm counts (Giovambattista *et al*., 2003; Nayanatara *et al*. 2008; Igwebuike *et al*., 2011).

## CONCLUSION

The results of the present investigation have shown that MSG increased the body weight and reduced the testis weight of rats, in addition to adversely affecting testicular histology. MSG also decreased sperm count, motility, sperm cells with morphology, and increased sperm cells with abnormal morphology. However, the intervention role of *A. hybridus* mitigated these effects.
